# Determining Falls Risk in People with Parkinson’s Disease Using Wearable Sensors: A Systematic Review

**DOI:** 10.3390/s25134071

**Published:** 2025-06-30

**Authors:** Maeve Bradley, Sarah O’Loughlin, Eoghan Donlon, Amy Gallagher, Clodagh O’Keeffe, John Inocentes, Federica Ruggieri, Richard B. Reilly, Richard Walsh, Tim Lynch, Daniel G. Di Luca, Conor Fearon

**Affiliations:** 1 Dublin Neurological Institute, Mater Misericordiae University Hospital, D07 R2WY Dublin, Ireland; 2 School of Medicine, Trinity College Dublin, D02 PN40 Dublin, Ireland,; 3 Department of Neurology, Washington University in St. Louis, St. Louis, MO 63130, USA

**Keywords:** sensors, Parkinson’s, falls risk

## Abstract

A prior history of falls remains the strongest predictor of future falls in individuals with Parkinson’s disease (PD). There are limited biomarkers available to identify falls risk before falls begin to occur. The aim of this review is to investigate if features associated with falls risk may be detected by wearable sensors in patients with PD. A systematic search of the MEDLINE, EMBASE, Cochrane, and Cinahl databases was performed. Key quality criteria include sample size adequacy, data collection procedures, and the clarity of statistical analyses. The data from each included study were extracted into defined data extraction spreadsheets. Results were synthesized in a narrative manner. Twenty-four articles met the inclusion criteria. Of these, twelve measured falls prospectively, while the remaining relied on retrospective history. The definition of a “faller” varied across studies. Most assessments were conducted in a clinical setting (18/24). There was considerable variability in sensor placement and mobility tasks assessed. The most common sensor-derived measures that significantly differentiated “fallers” from “non-fallers” in Parkinson’s disease included gait variability, stride variability, trunk motion, walking speed, and stride length.

## 1. Introduction

Parkinson’s disease (PD) is a progressive neurodegenerative disease characterized by tremor, bradykinesia, postural instability, and rigidity. Falls occur frequently in patients with PD; incidence rates vary from 35 to 90% for falls and from 18 to 65% for recurrent falls [1]. The consequences of these falls are considerable and play a major role in diminished quality of life. These include injuries [2], fear of falling [3], and reduced activity levels [4]. Falls are not limited to those with advanced Parkinson’s disease; they also frequently occur in early, untreated PD [5].

Among all clinical predictors, a prior history of falls remains the strongest predictor of future falls in PD [6]. Additional clinical risk factors include freezing of gait, cognitive impairment, reduced walking speed, and an inability to stand on one leg [7,8]. The National Parkinson Foundation’s Falls Task Force provided a comprehensive overview of clinical fall risk factors, reinforcing the consensus that a history of previous falls is the strongest predictor of future falls [9].

However, there is a lack of reliable objective methods for prospectively identifying individuals at risk of falling. Most clinical rating scales for gait and posture either perform sub-optimally for falls prediction or have been evaluated insufficiently [10]. Camera-based motion analysis has allowed further biomechanical evidence for gait parameters and falls risk, identifying increased head motion and reduced toe clearance in PD “fallers” [11]. However, this method requires stationary and often costly laboratory equipment [12].

Wearable sensors have emerged as promising tools for falls prediction. These devices can be embedded into clothes, wristbands, patches, or insoles. Inertial measurement units (IMUs) typically consist of an accelerometer (measures acceleration), a magnetometer (orientation), and a gyroscope (angle and angular velocity) [13]. Recent advances in miniaturization, battery life, and materials have improved sensor comfort, wearability, and long-term usability [14]. Wearable sensors can quantitatively assess and differentiate movement problems in people with PD [15]. They can accurately differentiate between PD and healthy controls, track progression of motor symptoms and detect falls and freezing of gait [15,16,17,18].

However, challenges remain, including managing large volumes of variable data, ensuring device reliability, and optimizing usability for both patients and clinicians. Artificial intelligence (AI) has been adapted to analyze physiological movement using machine learning and neural network technologies to detect patterns within large datasets. In combination with wearable sensors, AI provides advanced noise filtering algorithms which can help to distinguish relevant signals from motion artefacts or environmental noise, thereby enhancing the reliability of clinical data [19].

It is still unknown whether there is an ideal protocol for using wearable sensors in PD to reliably distinguish between “fallers” and “non-fallers”, or if any specific sensor-derived measurements hold the highest predictive value. There is also no consistent interpretation of what defines a “faller” or “recurrent faller” in PD. A better understanding of PD kinematic risk factors for falls could help identify those at risk and inform early interventions to mitigate this risk.

### Objective

Our objective is to determine the predictive accuracy of sensors in identifying PD “fallers” and to determine which specific sensor-derived parameters are most strongly associated with falls risk.

Furthermore, we intend to assess methodological differences across studies, including sensor type, placement, and analysis techniques.

## 2. Materials and Methods

A systematic review was conducted according to the PRISMA guidelines and in accordance with the *Cochrane Collaboration Handbook for Systematic Review of Interventions* [20]. The protocol was pre-registered (PROSPERO 2025 CRD420250640812).

### 2.1. Search Strategy

A systematic search of the MEDLINE, EMBASE, Cochrane, and Cinahl databases was performed with the search terms below. The last search was conducted on 31 March 2025. The search terms were as follows:

(“parkinson*” OR “PD”)
AND
((“wearable (device OR sensor OR technology OR system)”) OR “body worn (sensor OR device OR technology)” OR “insoles” OR “accelerometer” OR “gyroscope” OR “smartwatch” OR “pressure sensor” OR “kinematic”)
AND
(“falls” OR “falls (risk OR prediction OR forecasting OR count OR detection OR modelling)” OR “falling”)

In addition, the reference lists of all included articles were searched for additional relevant articles. No publication date restrictions were included. Articles that analyzed data from the same cohort were individually assessed to determine if outcome measures differed and were relevant to inclusion/exclusion criteria.

### 2.2. Eligibility Criteria

Original research articles published in English were included. Case studies, review/opinion articles, and conference abstracts were not included.

The defined population was adults with idiopathic PD as diagnosed by a movement disorder specialist. Articles were excluded if idiopathic PD was not the primary disorder (i.e., atypical Parkinson syndromes or other causes of Parkinsonism not included). We included participants at all stages of PD including, patients who were levodopa naïve, on oral medications, and on device-assisted therapies.

We included articles that used wearable sensors deployed in gait, posture, or balance assessments including in normal daily activities. Pressure mats and motion capture cameras were not included as these did not qualify as “wearable”.

Articles were included that determined the predictive accuracy of sensors in identifying PD “fallers”. No standardized definition of “fallers” or defined length of follow up of falls measurement was applied. Both retrospective and prospective measurement of falls were included. We excluded articles that did not record relevant outcomes with falls risk. Articles that assessed only freezing of gait were not included. Articles that used sensors only for falls detection (recognizing a fall event) rather than falls risk detection (evaluating the likelihood of falling) were not included.

### 2.3. Screening and Data Extraction

Two review authors (MB and SOL) independently screened the abstracts. A third author (CF) arbitrated in case of disagreements. The data from each included study was extracted into defined data extraction spreadsheets.

The following data was extracted: number of participants, participant characteristics, number and type of devices used, the anatomical location they were worn, and the locations and procedures in which the device was deployed (i.e., remote or in a laboratory/clinic environment). The primary outcome was dichotomous—“faller” vs. “non faller”—and predictive measures for outcome were extracted and summarized. Specific sensor-derived measures and their validity in differentiating “fallers” from “non-fallers” were identified. In particular, gait and stride variability, cadence, stride length, postural sway, harmonic ratio, foot/toe angle, and turning characteristics were recorded.

### 2.4. Data Synthesis

For each included study, outcomes related to fall risk prediction were synthesized using descriptive statistics and effect measures appropriate to the data type. In a standardized extraction form, we collected study characteristics, including first author, year of publication, study design, geographic location, assessment task, participant selection criteria, follow-up duration, and reported outcomes. Participant-level data, such as age, gender, disease duration, levodopa dosage and timing, cognitive function, MDS-UPDRS score, and Hoehn and Yahr stage, as well as sensor type, position, and testing parameters were also extracted.

For continuous variables (e.g., age, disease duration, UPDRS scores), effect measures included means and standard deviations, allowing for comparison between fallers and non-fallers. For categorical variables (e.g., gender, sensor type, presence of comorbidities), results were summarized using frequencies and percentages.

Due to heterogeneity in outcome reporting and study design, a meta-analysis was not performed. Instead, a qualitative synthesis was conducted. Data were presented in tables, narrative summaries, and visual graphs to facilitate the interpretation of trends and variability across studies.

### 2.5. Quality Assessment

Key quality criteria included sample size adequacy, data collection procedures, and the clarity of statistical analyses. JBI version 2020 was used as a critical appraisal tool. Two reviewers independently assessed the risk of bias. Disagreements were resolved through discussion or arbitration by a third reviewer (CF). Articles considered to have a high risk of bias were excluded. All included studies were assessed for potential risk of bias due to missing results, particularly arising from selective reporting or attrition

## 3. Results

### 3.1. Article Selection

**Figure 1:**
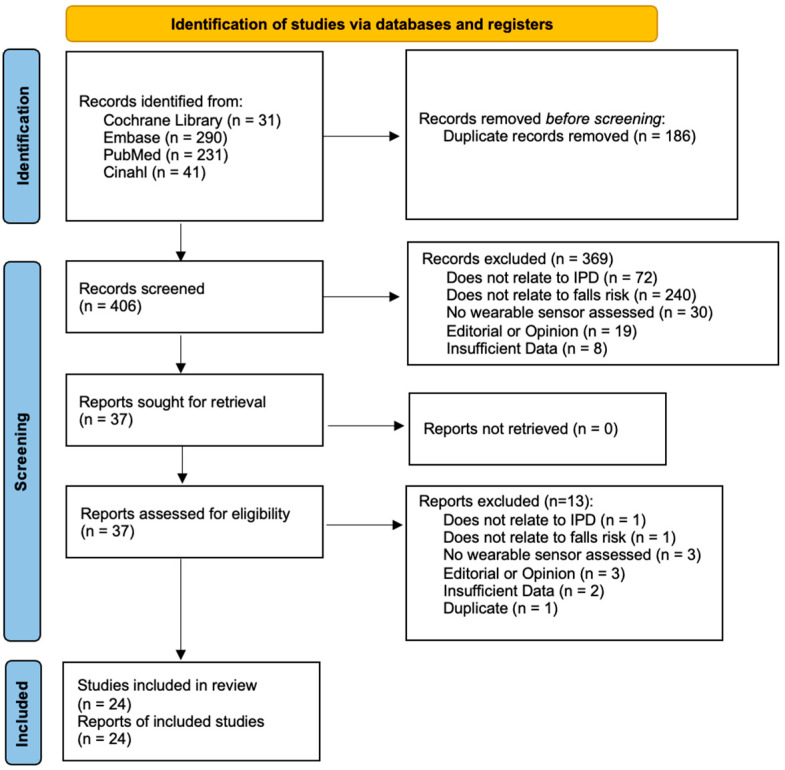
PRISMA flow diagram article selection.

A total of 406 abstracts were screened against the inclusion/exclusion criteria. Twenty-five articles proceeded to quality assessment, while one study was excluded due to risk of bias (see below). The primary reasons for exclusion included a wearable sensor not being assessed, PD not being the primary disorder, and falls risk association not being properly assessed (Figure 1).

One article included analyses of two separate datasets, namely “PD1” and “PD2” [21]. The PD1 dataset had been previously reported in a 2018 article included in this review [22]. Therefore, for our analysis, we exclusively assessed the PD2 dataset from the 2021 article.

Additionally, two articles used the same dataset but were both included in the review, as each reported different outcome measures [23,24].

### 3.2. Risk of Bias

Overall, the quality of the included studies was moderate. One study was excluded due to high risk of bias, stemming from a short follow-up, reliance on missteps rather than actual falls, and an inability of the sensors to distinguish between near-misses and true events [25].

Controlling for confounders in Parkinson’s disease is challenging due to its multifactorial nature. Many studies excluded individuals with severe comorbidities, such as cognitive impairment or mobility limitations. While this improved internal validity, it may reduce generalizability to higher-risk, real-world populations.

Most included studies were judged to have mild to moderate risk of bias. Technical issues, like sensor malfunctions accounted for some missing data, but there was no evidence of systematic exclusion or selective reporting affecting the overall synthesis. A total of 24 studies were included in the final review.

### 3.3. Study Design 

Of the twenty-four included articles, twelve were prospective in design, and the remaining twelve were cross-sectional (summarized in Table 1). The average number of participants in the studies was 71 (standard deviation (STD) 59, median 48, range 15–260). Overall, the 24 articles consisted of 1724 participants, of which 43% were defined as “fallers” by the respective authors (749/1724) (Supplementary Table S1). Falls measurement details are summarized below.

### 3.4. Participant Characteristics 

“Fallers” as defined by the respective authors were older (average age 68.5 (STD 2.5) vs. 66.1 (STD 2.2)). On average, “fallers” had greater disease severity as measured by higher MDS-UPDRS part III scores (average 35.4 vs. 29.1) and H&Y scores (average 2.4 vs. 1.9). Thirteen articles provided details on levodopa equivalent doses (LEDD) with a range of 400–1335 mg. No participants were reported to be levodopa naïve or on advanced therapies (Supplementary Table S1)

### 3.5. Task

Five articles assessed some variation on the timed up and go test. Four articles used wearable sensors in the patient’s home environment with application between 3–14 days. Nine articles assessed patients walking in the clinic at a self-selected speed for varying durations and lengths. Dual tasking was assessed in two cohorts. The primary objective of two studies was to assess the utility of an instrumented version of a standard quantitative assessment tool, namely the QTUG (Quantitative TUG) and imCTSIB (instrumented Modified Clinical Test of Sensory Interaction in Balance).

### 3.6. Sensor Details 

Accelerometers and gyroscopes were the most commonly used wearable sensors. Eleven articles used a single sensor application site and eleven used multiple sites (Supplementary Table S2).

### 3.7. On/Off Designations

Of the twenty-four articles, ten articles assessed patients in the “on” state and five in the “off” state. Three articles assessed patients in both “on” and “off” states, and in the reminder of articles “on/off” status was not specified. In the articles that assessed PD patients in both states, “off” parameters were more informative compared to “on” parameters. Tsai et al. found that dopaminergic therapy can improve clinical functional scores but worsens balance-related measures [29].

### 3.8. Falls Measurement 

Falls were measured prospectively in eight articles, retrospectively in twelve, and both prospectively and retrospectively in four. The maximum duration of falls measurement was 60 months (mean 6 months, median 12 months). The prospective range of falls measurement was 3–60 months, and the retrospective range was 6–12 months. The definition of a “faller” was not consistent (range > 2 falls in 6 months—>/= 1 fall/12 months). Falls measurements were subjective in all articles, either self-reported via phone or interview or captured by a falls diar (Supplementary Table S3)

### 3.9. Sensor-Derived Parameters Most Strongly Associated with Falls 

**Figure 2:**
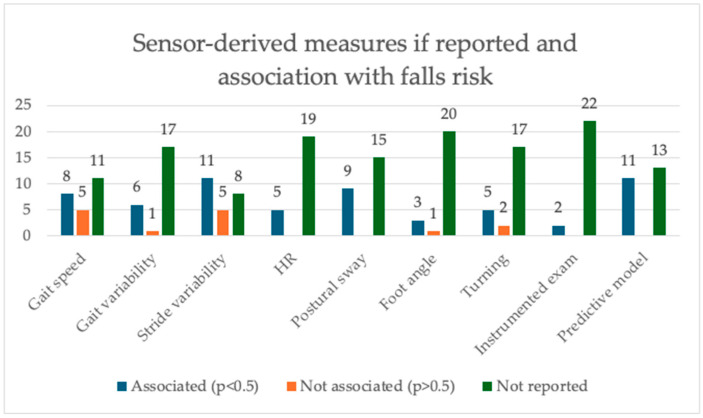
Bar chart showing the number of articles reporting on specific sensor-derived measures and ability to distinguish between “fallers” and “non fallers”.

Findings varied regarding which sensor-derived measure was most strongly associated with fallers compared to non-fallers, and the measures reported were not consistent across studies (Figure 2, ST).

#### 3.9.1. Walking Speed

Thirteen articles measured and reported on walking speed. Eight found reduced walking pace significantly distinguished “fallers” from “non-fallers” whereas five found no significant association.

#### 3.9.2. Gait and Stride Variability

Seventeen articles measured and reported on gait or stride variability. Six articles reported that increased gait variability was positively associated with falls risk, while eleven found stride variability (either timing or length) to be positively associated with falls risk. Five articles found no association between increased stride variability and falling status and one article found no association with gait variability.

#### 3.9.3. Gait Smoothness (Harmonic Ratio) and Postural Sway

The harmonic ratio (HR) is a measure of trunk acceleration commonly used as marker of gait smoothness. Five articles reported on this, and all five demonstrated that reduced HR was significantly associated with PD “fallers” (*p* < 0.001—*p* < 0.02). Nine articles reported on postural sway, and all nine demonstrated that increased or variability postural sway was significantly associated with the “faller” category; this included sway both anteroposteriorly and laterally.

#### 3.9.4. Foot Angle

Four articles reported on foot strike angle, with three finding a significant association with PD “fallers”.

#### 3.9.5. Turning

Seven articles reported on turning markers including velocity, duration, step count, and variability. Five found significant associations with PD “fallers”.

#### 3.9.6. Instrumented Exams

Two articles found that standardized mobility assessments’ ability to predict falls risk was enhanced by using sensor-derived measurements with the clinical measurements (QTUG and imCTSIB).

#### 3.9.7. Modeling

Eleven articles used clinical and sensor-derived mobility measures to develop falls prediction models for PD, demonstrating moderate to excellent predictive value (area under the curve 0.838—>0.988). In all models, a combination of clinical and sensor measurements outperformed clinical measurements alone.

## 4. Discussion

Falls risk in Parkinson’s disease is influenced by a complex interplay of motor, cognitive, and sensory impairments, making accurate prediction and prevention challenging [9,46,47]. While wearable technology shows promise in falls risk prediction, further validation and refinement are required to address variability. Key findings in this review suggest slower walking velocity, gait/stride variability, shorted stride length, reduced gait smoothness, and increased head and pelvis motion are associated with “fallers”. However, heterogeneity in studied population, sensor placement, and gait and posture assessment tasks complicate the generalization and comparison of results.

This year, the Gait Advisors Leading Outcomes for Parkinson’s (GALOP) committee provided recommendations for a minimum set of gait measures to be adopted in projects evaluating PD [48]. These includes (1) one continuous gait task of at least 1 min in duration, (2) over a path of 10 m in length (allowing 1 m at each end for a safe turn), and (3) with participants performing 180 degrees turns at each end. Only two included articles fulfilled these criteria [40,49]. Both studies incorporated dual tasking, a recommendation also endorsed by the GALOP committee. Although 10 of the 25 articles did include measurements of walking at a self-selected pace, the path length varied.

**Figure 3:**
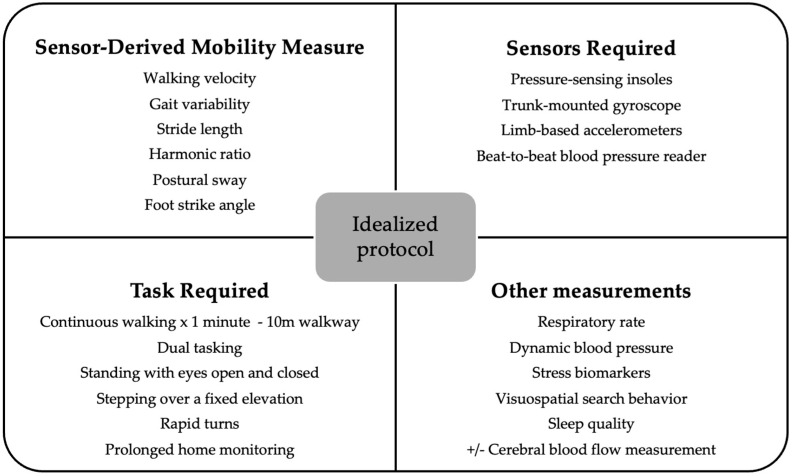
Suggested idealized protocol for sensor-based evaluations of falls risk in PD.

An idealized study protocol utilizing wearable sensors in PD has yet to be clearly defined. Optimal protocols may vary depending on the specific primary outcomes of a given study. From this review, we would recommend ensuring walking velocity, gait variability, stride length, harmonic ratio, postural sway, and foot strike angle are evaluated (Figure 3). Thus, clinical assessments using wearable sensors should incorporate a comprehensive sensor suite, including pressure-sensing insoles, trunk-mounted gyroscopes, limb-based accelerometers, and device-based measurements of confounders e.g., orthostatic hypotension. Assessing patients in “on” and “off” states is also optimal but logistically challenging 

Participants should also undergo a detailed clinical evaluation to identify and control for potential confounding factors, such as muscle weakness, musculoskeletal disorders, proprioceptive impairments, and visual deficits. To better distinguish PD-specific changes from those associated with aging, the inclusion of an age-matched healthy control group is strongly recommended. In addition to baseline clinical assessments, and the GALOP gait analysis [48], freezing of gait provocation protocols (rapid 360-degree turns in place [50]) and postural stability assessments (e.g., Mini-BESTest) should be considered.

For prolonged home monitoring, the selection of wearable sensors must be rationalized. Additional sensor modalities, such as those measuring respiratory rate, dynamic blood pressure, stress biomarkers, visuospatial search behavior, cerebral function/blood flow, and sleep quality, may provide complementary insights, but their inclusion should be guided by the specific aims and feasibility of the study.

This review was consistent with others in identifying slower walking speed and reduced stride length as markers of falls risk [9,51,52]. However, walking speed and its effect on fall risk is complex. Walking speed is determined by both the number of strides taken per unit of time (cadence) and the average stride length [53]. There is a suggestion that gait slowing in PD is primarily attributed to a reduction in stride length rather than a decrease in cadence [54,55], but they may coincide [15]. In this review, stride length was more frequently identified as a marker of falls risk than cadence. Additionally, individuals with PD, particularly those with postural instability, may consciously reduce their walking speed as a strategy to minimize fall risk.

In older adults, faster walking speeds have been associated with an increased risk of falls; however, the association is non-linear [42,53,56,57]. One study included in this review demonstrated that imposed faster walking speeds in individuals with PD led to decreased gait stability, while poorer stability was observed for all participant groups at walking speeds that were slower than preferred [58]. Further research is needed to determine whether reduced walking speed itself is an independent risk factor—separate from stride length—or if it primarily reflects a compensatory behavioral adaptation. Addressing this question would require sensor-based gait assessments. Regardless, reduced walking speed may serve as a valuable tool for identifying PD patients at risk of falls, as demonstrated in prior reviews [8,9,51,52,59].

In a recent review, gait variability, specifically stride-time variability, was identified as the only PD gait biomarker sensitive across four contexts of use (disease susceptibility/risk, progression, exercise response, and fall risk/prognosis), reflecting sensitivity but potentially poor specificity [60]. Gait variability in older adults is a well-documented risk factor for falls, with increased variability associated with a higher likelihood of falling [61,62,63,64]. However, gait variability was not identified as a strong predictor of falls risk in another meta-analysis of gait kinematics and falls risk in PD [65]. Levodopa can effectively improve stride length but does not fully restore gait variability and rhythmicity [64,66,67]. This underscores the need for multimodal approaches in gait rehabilitation, beyond just dopaminergic therapy.

### Limitations

There was limited consistency across the studies in terms of the task and sensor protocol. Differences in sensor types, placement, and the nature of the tasks assessed limited the comparability of results across studies. Upper body and foot measures were often excluded. Similarly, there was a lack of participant-level data to ensure that selected PD populations were similar for comparison, while outcome measures (including definition of a “faller”) varied. Furthermore, all articles included relied on some method of self-reporting falls, which is subject to recall bias and underreporting [68,69]. No study employed automated fall detection, although they also have false positives and negatives [70].

PD symptoms and, therefore, falls risk fluctuate diurnally and with medication cycles, so a single assessment as deployed by many studies may not capture an accurate falls risk profile. Although some studies utilized home-based monitoring, many relied on structured clinical assessments. Real-world gait and balance assessments may provide more ecologically valid indicators of fall risk. Many studies did not clearly specify whether participants were in the “on” or “off” medication state during the assessment.

## 5. Conclusions

Wearable sensors hold strong potential for early fall risk detection in PD. In this review, PD patients at risk of falling showed a profile of abnormalities detectable by wearable sensors, e.g., slower and more variable gait, increased sway, and often overall greater motor impairment. This review included twenty-four articles exploring wearable sensors in predicting falls risk in PD. We have highlighted significant variability in protocols and measurement standards across studies. Nonetheless, sensor-augmented assessments consistently outperformed traditional clinical evaluations in predicting fall risk, but models combining both performed superiorly again. Further work is needed to determine the minimum sensor setup and duration of application that still yields high predictive accuracy. Furthermore, there is a need for a unified definition of “faller” to standardize outcome measures.

## Figures and Tables

**Table 1:** Study design and outcome.

First Author	Year	Study Design	Study Location	Total PD	HC	Task	Outcome
Smulders, Katrijn [26]	2012	Prospective cohort	Clinic	260		Walk along a 10 m walkway with and without a dual task	Walking speed (*p* = 0.041) and stride length (0.012) associated with “fallers”. Deterioration of gait under dual task conditions not associated with falls risk.
Latt, Mark D [27]	2009	Prospective cohort	Clinic	113		Physiological Profile Assessment (PPA). Stand as still as possible on the floor and a foam rubber mat with eyes open and eyes closed. Leaning balance	Abnormal axial posture (*p* = 0.05) and poor coordinated stability (*p* = 0.015) associated with “fallers”. Model accurately identified “fallers”.
Ullrich, Martin [28]	2023	Prospective cohort	Both	35		Usual activites (home) × 2 weeks + unsupervised 4 × 10 MWT three times a day	Gait variability (*p* < 0.05), stride length, IC angle, max foot lift, and walking speed (*p* < 0.001) associated with fallers. Falls risk better predicted with real world gait data.
Tsai, Chang-Lin [29]	2022	Prospective cohort	Clinic	95		Standing with eyes open in the off- and on-medication states	Length and velocity of postural sway (*p* = 0.013 (cluster) was the highest predictor. Dopaminergic therapy improved clinical scores but worsened balance.
Shah, Vrutangkumar [24]	2023	Prospective cohort	Home	34		Usual activities (home) × 1 week	Stride time variability (*p* = −0.004), toe out angle variability (*p* < 0.001), angle foot midswing (*p* = 0.002), and turn velocity (*p* = 0.003) were the most selected discrimaters.
Ma, Lin [30]	2022	Prospective cohort	Clinic	51		TUG extended to 7 m	Gait variability (*p* = 0.010). RoMtrunk sagittal (*p* = 0.002), stride length variability (*p* = 0.039), and swing phase variability (0.023) were risk factors for falls.
Sturchio, Andrea [31]	2021	Prospective cohort	Both	26		Usual activities (home) + lying to standing + TUG + 2MWT + sway eyes open/closed	Waist sway (*p* < 0.01), jerkiness, and centroidal frequency
Greene, Barry R [22]	2018	Prospective cohort	Clinic	15		TUG monthly × 6 months	QTUG (73.3% accuracy predicting falls at 90 days)
Cole, Michael H [32]	2017	Prospective cohort	Clinic	79	82	4 self-paced and barefoot walking trials along a 9 m-long firm walkway.	Increased trunk flexion (*p* = 0.01), lateral head (*p* = 0.009) and trunk motion (*p* = 0.008), and increased trunk muscle activiation on EMG (*p* < 0.05) associated with fallers.
Weiss, Aner [33]	2014	Prospective cohort	Home	107		Usual activities (home) × 3 days	Gait variability (AP) (*p* = 0.012), lower stride regularity (*p* = 0.018), and less smooth gait pattern (lower HR) (*p* = 0.011) associated with fallers.
Hoskovcová, Martina [34]	2015	Prospective cohort	Clinic	45	22	TUG extended to 7 m	Stride variability (off) (*p* < 0.01) and cadences (off) (*p* < 0.01) and gait speed (*p* < 0.01) were the most significant predictors.
Sotirakis, Charalampos [35]	2024	Prospective cohort	Clinic	104 *		Walk for 2 min on 15 m corridor. Stand still with eyes closed.	Gait variability, postural sway acceleration variability, and stride length (*p* < 0.01 each) were the most significant predictors.
Castiglia, Stefano Filippo [36]	2021	Cross-sectional	Clinic	55	30	Walk at a self-selected speed	Reduced harmonic ratio AP (*p* = 0.004), increased pelvic obliquity (*p* = 0.024), and increased pelvic rotation (*p* = 0.040) associated with fallers.
Latt, M. D. [37]	2009	Cross-sectional	Clinic	66	33	Walk at a self-selected speed along a 20 m corridor	Walking speed (*p* < 0.01), step timing variability (*p* < 0.01), and reduced HR (vertical *p* < 0.001) were the most significant predictors.
Del Din, Silvia [38]	2019	Cross-sectional	Home	170	172	Usual activities (home) × 1 week	Lower step velocity and length (*p* < 0.05), and stride length variabilty (*p* = 0.004) were the most significant predictors.
Hubble, Ryan P [39]	2016	Cross-sectional	Clinic	29		TUG × 5 + 6MWT + retropulsion	Reduced HR (rhymicity) of the head and trunk (*p* < 0.02) was the most significant predictor.
Araújo, Hayslenne A G O [40]	2023	Cross-sectional	Clinic	127		Walking at self-selected pace along a 10 m corridor for 2 min with and without dual task.	Foot strike angle, variability of trunk tranverse ROM, stride variability, and turn duration/steps (*p* < 0.05 for each) were the most significant predictors.
Shah, Vrutangkumar V [23]	2022	Cross-sectional	Both	34		3 min walk test at natural pace in on and off state + usual activities (home) × 1 week	Turn velocity, number of steps in turn, and variability in gait speed (in “off” state) (*p* < 0.03) were the most significant predictors. No sig difference in “on” state.
Schaafsma, Joanna D [41]	2003	Cross-sectional	Clinic	32		80 m walking test in the “off” and “on” state	Stride time variability (*p* < 0.009) was the most significant predictor.
Cole, Michael H [42]	2017	Cross-sectional	Clinic	20	10	Walking on a treadmill at 70%, 100%, and 130% of preferred speed.	Reduced HR, reduced speed, and stride length were the most significant predictors.
Freeman, Lynn [43]	2018	Cross-sectional	Clinic	26		Sensory organisation test and modified clinical test of sensory integration	I-mCTSIB (instrumented exam) may distinguish between fallers and nonfallers (*p* = 0.04)
Plotnik [44]	2011	Cross-sectional	Clinic	30		Walking along 20 m walkway with and without dual tasking	Walking speed, gait variability, and asymmetry. Larger DT effect in fallers.
Greene, Barry R [21]	2021	Cross-sectional	Clinic	27	1015	TUG	Predictive model: mean R2 value 0.43, mean error 0.42, mean correlation 30% across 2 data sets. PD2: mean stride length and no. strides in turn.
Vitorio, Rodrigo [45]	2023	Cross-sectional	Clinic	144		Walk at comfortable pace for 2 min. Stand for 30 s in 3 different condition; firm surface, eyes either open or closed; foam surface, eyes open.	Turn variability (*p* = 0.04), step duration (*p* = 0.007) and stride Length variability (*p* = 0.002) and trunk transverse ROM (*p* = 0.11) were the most significant predictors.

* Fall naïve, NR: Not reported, HC: healthy control, TUG: timed up and go test (individuals start seated, walk 3 m, turn around, and sit back down. The time is recorded), MWT: meter walk test. PPA: The Physiological Profile Assessment, mCTSIB: Modified Clinical Test of Sensory Interaction in Balance.

## Data Availability

The original contributions presented in this study are included in the article/Supplementary Materials. Further inquiries can be directed to the corresponding author.

## Supplementary Materials

The following supporting information can be downloaded at: https://www.mdpi.com/article/10.3390/s25134071/s1, Supplementary Table S1: Summary of participant characteristics; Supplementary Table S2: Sensor details; Supplementary Table S3: Falls measurement; Supplementary Table S4: Summary of sensor derived measures and association with “fallers”.

## Abbreviations

The following abbreviations are used in this manuscript:

PD	Parkinson’s disease
HR	Harmonic ratio
TUG	Timed up and go
