# Supplementary material for: Determining Falls Risk in People with Parkinson’s Disease Using Wearable Sensors: A Systematic Review
Source: Sensors (Basel). 2025 Jun 30;25(13):4071. doi: 10.3390/s25134071 (PMC12251786; doi:10.3390/s25134071)
Supplement: Supplementary file 1 [file sensors-25-04071-s001.zip › sensors-3590160-supplementary.pdf]

Table S1. Summary of participant characteristics.

Figure .	Year	Category	n (% female)	Mean Age (SD)	Mean Total UPDRS (SD) (part III=*)	Mean H&Y (Mean)	Disease Duration (Years, SD)	LEDD total score mg/day (SD)	Cognition (MoCa) (SD)	On/Off State
Green, Barry R.	2018	Fallers	8 (50.0)	66.4 (6.8)	16.0 (9.6)*	NR	NR	NR	NR	Unknown
		Nonfallers	6 (16.7)	68.7 (7.9)	14.8 (10.9)*	NR	NR	NR	NR	
		Total	15 (33.3)	67.4 (7.1)	15.5 (9.8)*	NR	NR	NR	NR	
Del Din, Silvia.	2019	Fallers	155 (38.0)	71.6 (6.4)	31.4 (13.2)*	2.5	NR	NR	NR	On
		Nonfallers	15 (26.7)	64.5 (8.6)	28.6 (5.7)*	2	NR	NR	NR	
		Total	170 (37.0)	70.9 (6.9)	31.1 (12.7)*	2.4	NR	NR	NR	
Hubble, Ryan P	2016	Fallers	23 (NR)	NR	NR	NR	NR	NR	NR	Unknown
		Nonfallers	7 (NR)	NR	NR	NR	NR	NR	NR	
		Total	29 (27.6)	64.7 (6.4)	14.4(11.5)	1.7(0.7)	6.7	618.3 (432.1)	91.7 (6.1) [1]	
Vitorio, Rodrigo	2023	Fallers	54 (38.9)	70.8 (7.6)	77.0 (22.2)	2.5 (0.8)	7.6 (5.9)	645.6 (449.1)	25.7 (3.6)	Off
		Nonfallers	90 (29.6)	67.3 (8.1)	61.9 (17.5)	2.1 (0.5)	5.3 (4.1)	577.8 (337.2)	25.9 (3.4)	
		Total	144 (32.6)	68.6 (8.1)	67.6 (20.7)	2.3 (0.7)	6.2 (5.0)	603.2 (382.9)	25.8 (3.5)	
Ullrich, Martin		Fallers	10 (40.0)	64.5 (8.2)	21.9 (9.1)*	2.9 (0.5)	NR	NR	NR	On
	2023	Nonfallers	25 (20.0)	63.5 (8.4)	12.9 (6.2)*	2.3(0.5)	NR	NR	NR	

		Total	35 (25.7)	63.8 (8.2)	15.5 (8.1)*	2.5 (0.6)	NR	NR	NR	
Greene, Barry R	2021	Fallers	4	NR	NR	NR	NR	NR	NR	Unknown
		Nonfallers	11	NR	NR	NR	NR	NR	NR	
		Total	27 (34.63)	64.9 (7.3)	22.6 (10.3) *	NR	NR	NR	NR	
Ma, Lin	2022	Fallers	14 (42.9)	65.2 (4.9)	37.9 (13.6)*	3	10.14(3.9)	412.5 (NR)	27 (NR)	On
		Nonfallers	37 (32.4)	65.9 (9.4)	31.9 (12.3)*	2	7.24(4.24)	618.8 (NR	26 (NR)	
		Total	51 (35.3)	65.7 (8.4)	33.6 (13.5)*	2.4	8.0 (4.4)	593.0 (364.8)	26.1 (3.4)	
Sturchio, Andrea	2021	Fallers	14 (28.6)	74.5 (12.1)	42.6 (14.6)*	NR	NR	1079 (NR)	NR	On
		Nonfallers	12 (25.0)	70.2 (7.6)	37.2 (11.7)*	NR	NR	850 (NR)	NR	
		Total	26 (26.9)	72.5 (9.9)	40.1 (13.4)*	NR	NR	973.3 (NR)	NR	
Sotirakis, Charalampos	2024	Fallers	23 (39.0)	71 (6.5)	NR	NR	8.3	NR	27 (3)	Unknown
		Nonfallers	74 (43.2)	65 (9.8)	NR	NR	2.5	NR	27 (3)	
		Total	97 (42.2)	66.42 (9.5)	NR	NR		NR	27 (3)	
Freeman, Lynn	2018	Fallers	5 (NR)	NR	NR	NR	NR	NR	NR	On
		Nonfallers	21 (NR)	NR	NR	NR	NR	NR	NR	
		Total	26 (NR)	66.0 (6.9)	32.7 (13.5)*	2 (median)	5.0 (4.2)	NR	NR	
Araújo, Hayslenne A G O	2023	Fallers	31 (NR)	70.73 (7.1)	39,42 (15.0)*	2.27(0.6)	7.18(6.3)	NR	26.5 (2.9)	On
		Nonfallers	96 (NR)	68.93 (8.0)	32.43 (16.4)*	1.99(0.7)	5.28 (4.3)	NR	27.3 (2.5)	

		Total	127 (NR)	69.6 (7.67)	35.2 (16.2)*	2.1 (0.7)	6.04 (5.2)	NR	27.0 (2.6)	
Shah, Vrutangkumar V	2022	Fallers	17 (NR)	68.7 (11.1)	On 32.7 (9.9) Off 46.2 (10.0)	On 2.2 (0.5) Off 2.3 (0.6)	9.2 (4.6)	1128.1 (533.2)	26.9 (2.9)	Both Specified
		Nonfallers	17(NR)	66.8 (6.6)	On 29.5 (8.5) Off 43.9 (11.3)	On 2 (0) Off 2.1 (0.2)	7.3 (5.6)	1541.9 (342.5)	26.9 (2.4)	
		Total	34 (NR)	67.8 (8.9)	On 31.1 (9.2) Off 68.1 (10.7)	On 2.1 (0.3) Off 2.2 (0.4)	16.5 (5.1)	1335 (437.9)	26.9 (2.7)	
Weiss, Aner	2014	Fallers	40 (35)	66.5 (8.21)	33.4 (11.41)*	2.9 (0.8)	6.08 (4.02)	400.2 (353.6)	29.0 (1.6)	Off
		Nonfallers	67 (19.4)	64 (9.76)	40.15 (13.35)*	2.4 (0.5)	5.15 (3.08)	454.7 (341.8)	28.3 (2.2)	
		Total	107 (25.2)	64.9 (9.3)	37.6 (13.0)*	2.6 (0.7)	5.5 (3.5)	434.3 (345.6)	28.5 (2.0)	
Castiglia, Stefano Filippo	2021	Fallers	19	NR	NR	NR	NR	NR	NR	Unknown
		Nonfallers	36	NR	NR	NR	NR	NR	NR	
		Total	55 (29)	71.2 (4.7)	39.5 (16.9)*	2.2 (0.8)	8.2 (5.4)	NR	NR	
Latt, M. D.	2009	Fallers	33 (54.5)	67 (2)	42(5)	3	9(2)	NR	N=8 score>27[2]	On
		Nonfallers	33 (54.6)	63 (4)	25(4)	1	7(2)	NR	N=17 score>27[2]	
		Total	66 (54.55	65 (3.2)	33.5 (4.5)	2	8 (2)	NR	N=25 score>25[2]	
		Fallers	NR	NR	NR	NR	NR	NR	NR	Off

Schaafsma, Joanna D	2003	Nonfallers	NR	NR	NR	NR	NR	NR	NR	
		Total	32 (28.1)	62 (7.5)	44.3 (16.0)*	2.9 (0.06)	9.6 (3.9)	673 (336)	28.1 (2.0)[2]	
		Fallers	10 (40)	69.3 (2.2)	22.6 (1.9) *	2.2 (0.2)	7.0 (1.7)	810.8 (147.8)	NR	
Cole, Michael H	2017	Nonfallers	10 (40)	66.5 (2.5)	13.1 (2.1) *	1.4 (0.2)	4.6 (0.6)	451.6 (102.9)	NR	Unknown
		Total	20 (40)	67.9 (2.4)	17.9 (2) *	1.8 (0.2)	5.8 (1.2)	***	NR	
		Fallers	27 (25.9)	65.6 (7.7)	On 18.6 (9.4). Off 31.7 (10.1)*	2.7 (0.3)	10.4 (4.0)	1265 (430.0)	24.0 (3.7)	
Hoskovcová, Martina	2015	Nonfallers	18 (22.2)	69.7 (6.3)	On 14.1 (6.5) Off 23.5 (6.3)*	2.3 (0.4)	9.8 (2.3)	915 (232.0)	24.6 (2.4)	Both Specified
		Total	45 (24.4)	67.2 (7.4)	On 16.8 (8.6) Off 28.4 (9.6)*	2.6 (0.4)	10.2 (3.4)	1124.9 (400.1)	24.2 (3.3)	
		Fallers	17 (NR)	68.7 (11.1)	On 32.7 (9.9) Off 46.2 (10.0)	On 2.2 (0.5) Off 2.3 (0.6)	9.2 (4.6)	1128.1 (533.2)	26.9 (2.9)	
Shah, Vrutangkumar V	2023	Nonfallers	17(NR)	66.8 (6.6)	On 29.5 (8.5) Off 43.9 (11.3)	On 2 (0) Off 2.1 (0.2)	7.3 (5.6)	1541.9 (342.5)	26.9 (2.4)	Both Specified
		Total	34 (NR)	67.8 (8.9)	On 31.1 (9.2) Off 68.1 (10.7)	On 2.1 (0.3) Off 2.2 (0.4)	16.5 (5.1)	1335 (437.9)	26.9 (2.7)	

		Fallers	48 (39.6)	69.1 (1.2)	38.2 (2.1)	2.1 (0.1)	7.2 (0.8)	763.0 (63.4)	94.0 (0.7)[1]	
Cole, Michael H	2017	Nonfallers	31 (29.0)	66.5 (1.4)	29.4 (1.8)	1.4 (0.1)	4.2 (0.6)	489.7 (60.7)	94.7 (0.9)[1]	On
		Total	79 (35.4)	68.1 (0.9)	34.7 (1.5)	1.9 (0.1)	6.1 (0.5)	655.7 (47.5)	94.3 (0.6)[1]	
		Fallers	51 (43.1)	68.3 (2.1)	75.0 (60.6)	2.5 (0.7)	3.8 (2.6)	80 (NR)	NR	
Latt, Mark D.	2009	Nonfallers	62 (43.5)	64.4 (2.7)	41.4 (48.3)	1.6 (0.7)	2.8 (2.3)	100 (NR)	NR	On
		Total	113 (43.3)	66.2 (2.5)	56.6 (54.1)	2.0 (0.7)	3.2 (2.5)		NR	
		Fallers	38.0 (55.3)	69.0 (9.5)	46.4 (16.9)	2.4 (1.0)	7.7 (5.0)	1146.9(684.2)	81.7 (12.4)[3]	
Tsai, Chang-Lin	2022	Nonfallers	57 (45.8)	67.6 (10.1)	34.2 (18.8)	1.9 (0.8)	4.0 (3.5)	604.3 (365.4)	83.5 (14.3)[3]	Off
		Total	95 (49.6)	68.4 (9.8)	39.1 (18.0)	2.1 (0.9)	5.5 (4.2)	821.3 (579.0)	82.8 (13.5)	
		Fallers	91 (37.4)	66.3 (7.5)	36.7 (9.4) *	1.99 (0.26)	NR	NR	27.8 (1.7)	
Smulders, Katrijn	2012	Nonfallers	171 (34.3)	64.6 (8.1)	32.7 (9.1) *	2 (0.26)	NR	NR	28.3 (1.5)	Unknown
		Total	263 (35.4)	65.2 (7.9)	34.1 (9.4)*	1.99 (0.24)	NR	NR	28.2 (1.6)	
		Fallers	16 (31.2)	68.6 (6.7)	36.2 (10.8)	2.1 (6.9)	10.4 (5.5)	NR	NR	
Plotnik, Meir	2011	Nonfallers	14 (28.6)	62.8 (6.8)	32.7 (9.7)	2.1 (0.6)	8.2 (3.2)	NR	NR	On
		Total	30 (0.3)	65.9 (7.2)	34.6 (10.3)	2.1 (0.6)	9.4 (4.6)	NR	NR	

NR: Not reported, SD: Standard deviation, UPDRS: Unified Parkinson's disease Rating Scale, [1] Addenbrooke's Cognitive Exam,[2] Mini mental state exam,[3]Cognitive Abilities Screening Instrument

Table S2. Sensor details.

First Author	Year	Sensor Location	Type of sensor	Single vs. multiple	Duration of application
Araújo, Hayslenne A G O	2023	Lumbar spine, sternum, wrists, shins, feet	Accelerometer, Gyroscope and Magnetometer	Multiple	Single session
Castiglia,StefanoFilippo	2021	Lumbar spine	Accelerometer, Gyroscope and Magnetometer	Single	Single session
Cole, Michael H	2017	Head and trunk	Accelerometer and Gyroscope	Multiple	Single session
Cole, Michael H	2017	EMG superficial trunk and VICON camera	Surface EMG and VICON (video camera)	Multiple	Single session
Del Din, Silvia	2019	Lumbar spine	Accelerometer	Single	7 days
Freeman, Lynn	2018	Lumbar spine	Accelerometer	Single	Single session
Greene, Barry R	2018	Shin	Accelerometer and Gyroscope	Single	Monthly TUG x 6months

Greene, Barry R	2021	Shin	Accelerometer and Gyroscope	Single	Four sessions
Hoskovcová, Martina	2015	NR	Accelerometer and Gyroscope	NR	Single session
Hubble, Ryan P	2016	Head and thoracic spine (T10)	Accelerometer	Multiple	Single session
Latt, M. D.	2009	Helmet and sacrum	Accelerometer	Multiple	Single session
Latt, Mark D.	2009	NR	Accelerometer	NR	Single session
Ma, Lin	2022	Legs, trunk and arms	Accelerometer and Gyroscope	Multiple	Single session
Plotnik	2011	Insoles	Force sensitive insoles	Single	Single session
Schaafsma, Joanna D	2003	Insoles	Force sensitive insoles	Single	Single session
Shah, Vrutangkumar V	2022	Top of foot and lumbar back	Accelerometer, Gyroscope and Magnetometer	Multiple	7 days
Shah, Vrutangkumar V	2023	Top of foot and lumbar back	Accelerometer, Gyroscope and Magnetometer	Multiple	7 days
Smulders, Katrijn	2012	Lower back	Accelerometer	Single	Single session
Sotirakis, Charalampos	2024	Trunk, wrist, lumbar spine, feet	Accelerometer, Gyroscope and Magnetometer	Multiple	Single session
Sturchio, Andrea	2021	Feet, wrists, sternum, and lumbar spine	Accelerometer, Gyroscope and Magnetometer	Multiple	Single session
Tsai, Chang-Lin	2022	Insoles	Force sensitive insoles	Single	Single session
Ullrich, Martin	2023	Insoles	Force sensitive insoles	Single	14 days

Vitorio, Rodrigo	202 3	Sternum, lumbar spine, wrists, shins and feet	Accelerometer, Gyroscope and Magnetometer	Multiple	Single session
Weiss, Aner	201 4	Lumbar spine	Accelerometer	Single	3 days

EMG: Electromyography, NR: Not reported

Table S3. Falls measurement.

First Author	Year	Falls measurement	How falls measured	Duration	Definition of faller
Hoskovcová, Martina	2015	Both	Falls diary monthly	6months	>/= 1 /6months
Shah, Vrutangkumar V	2023	Both	Self-reported + email every 2 weeks	12 months	>/=1/6months
Tsai, Chang-Lin	2022	Both	Self-reported - 3montly	12months	>= 1/12months
Weiss, Aner	2014	Both	Self-reported for year prior and prospectively by monthly calendar for 1 year	12months	>/=1/12months
Cole, Michael H	2017	Prospective	Falls diary monthly	12months	>/=1/12months
Greene, Barry R	2018	Prospective	Weekly falls diary	6months	>/= 1/6months
Latt, Mark D.	2009	Prospective	Monthly calendars with phone calls	12months	>/=1/12months
Ma, Lin	2022	Prospective	Monthly phone calls	6 months	>/=1/6months
Smulders, Katrijn	2012	Prospective	Monthly with automated telephone system + verified with personal call	12months	> 1/12months
Sotirakis, Charalampos	2024	Prospective	Interview at visits 3monthly for 2 years, then recorded by telephone and in person	60 months	NR
Sturchio, Andrea	2021	Prospective	Falls diary	6 months	>/= 1/6months
Ullrich, Martin	2023	Prospective	Paper based falls diary	3 months	>/= 1/3months
Araújo, Hayslenne A G O	2023	Retrospective	Self-reported	NR	>/=2/12months

Castiglia, Stefano Filippo	2021	Retrospective	Self-reported	12months	>/=1/12months
Cole, Michael H	2017	Retrospective	Self-reported	12months	>/=1/12months
Del Din, Silvia	2019	Retrospective	Self-reported	6months	>/=2 in 6months
Freeman, Lynn	2018	Retrospective	Self-reported	6 months	>/=2 /6months
Greene, Barry R	2021	Retrospective	Self-reported	12 months	>/=1/12months
Hubble, Ryan P	2016	Retrospective	Self-reported	12months	>/=1/12months
Latt, M. D.	2009	Retrospective	Self-reported	12months	>/=1/12months
Plotnik	2011	Retrospective	Self-reported	12months	>/=1/12months
Schaafsma, Joanna D	2003	Retrospective	Part 13 UPDRS	NR	"Any falls"
Shah, Vrutangkumar V	2022	Retrospective	Self-reported	6 months	>/=1/6months
Vitorio, Rodrigo	2023	Retrospective	Self-reported	6months	>/=1/6months

NR: Not reported

Table S4. Summary of sensor derived measures and association with "fallers".

First Author	Year	Gait variability (overall)	Stride Length	Stride variability	Gait smoothness (HR)	Walking speed	Postural sway	Turning	Foot strike angle	Predictive model
Castiglia, Stefano Filippo	2021	NR	NR	NR	+	NR	NR	NR	NR	
Latt, M. D.	2009	NR	-	+ timing	+	+	NR	NR	NR	
Del Din, Silvia	2019	NR	+	+ length	NR	+ lower step velocity	NR	NR	NR	
Hubble, Ryan P	2016	NR	NR	NR	+	NR	NR	NR	NR	
Smulders, Katrijn	2012	NR	+	-	NR	+	NR	NR	NR	
Latt, Mark D.	2009	NR	-	-	NR	-	+	NR	NR	Correctly classified 77% of fallers
Araújo, Hayslenne A G O	2023	NR	+	+	NR	NR	+ transverse		+	AUC 0.85
Shah, Vrutangkumar V	2022	+	-	-	NR	+	NR	+	-	
Ullrich, Martin	2023	+	+	NR	NR	+	NR	NR	+	Accuracy 75%
Tsai, ChangnLin	2022	NR	NR	NR	NR	NR	+ length and velocity	NR	NR	AUC 0.90
Shah, Vrutangkumar V	2023	NR	NR	+ timing	NR	NR	NR	+ velocity	+	AUC 0.94

Schaafsma, Joanna D	2003	NR	NR	+ timing	NR	NR	NR	NR	NR	
Ma, Lin	2022	+	-	+ length	NR	NR	+ sagittal	NR	NR	AUC 0.838
Cole, Michael H	2017	NR	+	-	+	+	+ AP acceleratio-	NR	NR	
Freema-, Lynn	2018	NR	NR	NR	NR	NR	NR	NR	NR	
Sturchio, Andrea	2021	NR	-	NR	NR	-	+	-	NR	AUC 0.87
Greene, Barry R	2018	NR	NR	NR	NR	NR	NR	NR	NR	
Cole, Michael H	2017	NR	-	NR	NR	-	+ lateral	NR	NR	
Weiss, Aner	2014	+	NR	+	+	NR	NR	NR	NR	
Plotnik	2011	+	-	+	NR	+	NR	NR	NR	
Greene, Barry R	2021	-	+	-	NR	-	NR	+ step count	NR	R2 model
Hoskovcová, Martina	2015	NR	NR	+	NR	+	NR	NR	NR	AUC 0.988
Sotirakis, Charalampos	2024	+	+	+ length	NR	-	+ sag, variability	-	NR	AUC 0.85*
Vitorio, Rodrigo	2023	NR	NR	+ length	NR	NR	+ transverse	+ variabilty	NR	AUC 0.94

* At 60months, +: Statistically significant difference between PD “fallers” vs “non-fallers”, -: No statistically significant difference between PD “fallers” vs “non-fallers”, NR: Not reported
